# Reduced Nrf2 expression mediates the decline in neural stem cell function during a critical middle‐age period

**DOI:** 10.1111/acel.12482

**Published:** 2016-04-20

**Authors:** Mandi J. Corenblum, Sneha Ray, Quentin W. Remley, Min Long, Bryan Harder, Donna D. Zhang, Carol A. Barnes, Lalitha Madhavan

**Affiliations:** ^1^ Department of NeurologyUniversity of ArizonaTucsonAZUSA; ^2^ Neuroscience and Cognitive Science Undergraduate ProgramUndergraduate Biology Research ProgramUniversity of ArizonaTucsonAZUSA; ^3^ Pharmacology and ToxicologyUniversity of ArizonaTucsonAZUSA; ^4^ Departments of Psychology & NeuroscienceUniversity of ArizonaTucsonAZUSA; ^5^ Evelyn F McKnight Brain InstituteUniversity of ArizonaTucsonAZUSA

**Keywords:** aging, oxidative stress, subventricular zone, redox, Nrf2, neural stem cells

## Abstract

Although it is known that the regenerative function of neural stem/progenitor cells (NSPCs) declines with age, causal mechanisms underlying this phenomenon are not understood. Here, we systematically analyze subventricular zone (SVZ) NSPCs, in various groups of rats across the aging spectrum, using *in vitro* and *in vivo* histological and behavioral techniques. These studies indicate that although NSPC function continuously declines with advancing age, there is a critical time period during middle age (13–15 months) when a striking reduction in NSPC survival and regeneration (proliferation and neuronal differentiation) occurs. The studies also indicate that this specific temporal pattern of NSPC deterioration is functionally relevant at a behavioral level and correlates with the decreasing expression of the redox‐sensitive transcription factor, Nrf2, in the NSPCs. When Nrf2 expression was suppressed in ‘young’ NSPCs, using short interfering RNAs, the survival and regeneration of the NSPCs was significantly compromised and mirrored ‘old’ NSPCs. Conversely, Nrf2 overexpression in ‘old’ NSPCs rendered them similar to ‘young’ NSPCs, and they showed increased survival and regeneration. Furthermore, examination of newborn Nrf2 knockout (Nrf2 −/−) mice revealed a lower number of SVZ NSPCs in these animals, when compared to wild‐type controls. In addition, the proliferative and neurogenic potential of the NSPCs was also compromised in the Nrf2−/− mice. These results identify a novel regulatory role for Nrf2 in NSPC function during aging and have important implications for developing NSPC‐based strategies to support healthy aging and to treat age‐related neurodegenerative disorders.

## Introduction

Aging is characterized by diminished adaptive capacity or plasticity in all tissues and organs. Core to this phenomenon is the reduced functioning of stem cells, which possess the ability to regenerate, repair, and protect cells to maintain optimal tissue function and homeostasis (Limke & Rao, 2003; Liu & Rando, 2011). The reduction in stem cell activity during aging sometimes occurs spontaneously, but often arises from deleterious changes in the local tissue environment. In particular, genetic predisposition along with environmental factors can produce oxidative stress, inflammation, reduced trophic factors, and somatic mutations in aged tissues, leading to dysregulation of intrinsic signaling pathways essential to stem cell survival and function (Mattson *et al*., 2002). Comprehending the cause of this age‐related deterioration of stem cell populations may hold the key to discovering approaches for promoting healthy aging and resisting a variety of age‐related disorders.

In the adult mammalian brain, neural stem/progenitor cells (NSPCs), which generate new neurons and glial cells, mainly inhabit two specialized niches: (i) the subventricular zone (SVZ) in the forebrain and (ii) the dentate gyrus (DG) of the hippocampus (Riquelme *et al*., 2008). In rodents, the SVZ is the larger pool of stem cells whose progeny migrate to the olfactory bulb (OB). As with other stem cells in the body, the capacity of SVZ NSPCs to self‐renew, proliferate, and generate new cells is significantly reduced with advancing age (Luo *et al*., 2006; Conover & Shook, 2011). More specifically, studies indicate that the SVZ NSPC niche is substantially contracted in size in aged animals with regeneration conserved only in the dorsolateral part of the structure (Luo *et al*., 2006). These elegant studies also detail the age‐related changes in the SVZ in terms of proliferation and effects on the various NSPC subtypes. Nevertheless, the temporal dynamics of how NSPC deteriorate during aging, and the molecular underpinnings of this process, are not clearly understood.

The goals of this study were to (i) systematically characterize the temporal pattern of NSPC regression during aging and (ii) investigate potential cell‐intrinsic mechanisms contributing to this phenomenon. In this context, our previous studies, as well as others, have identified the high expression of antioxidants as an innate characteristic of young NSPCs supporting their optimal survival, regeneration, and therapeutic function (Madhavan *et al*., 2006, 2007). In addition, it is also known that endogenous antioxidants govern cellular redox status, which is recognized as a fundamental regulator of NSPC function (Noble, 2006; Le Belle *et al*., 2011). Therefore, we specifically investigated the potential role of Nrf2 (nuclear factor erythroid‐derived 2‐like 2), a vital transcription factor regulating the cell's response to stress, in mediating the age‐related decline in NSPC regeneration (Motohashi & Yamamoto, 2004). First, we examined NSPC viability, regeneration, and Nrf2 expression, in several groups of rats across the aging continuum. Then, we assessed the effects of altered Nrf2 expression on rat NSPC function using RNA interference and overexpression assays, as well as in Nrf2 knockout mice. These studies identify a crucial time period during middle age, when there is a prominent reduction in NSPC survival and function, and indicate the reduced expression of Nrf2 as an important mechanism mediating this age‐related process.

## Results

### Characterization of NSPC survival and regenerative function during aging reveals a critical middle‐age period of vulnerability

**Figure 1:**
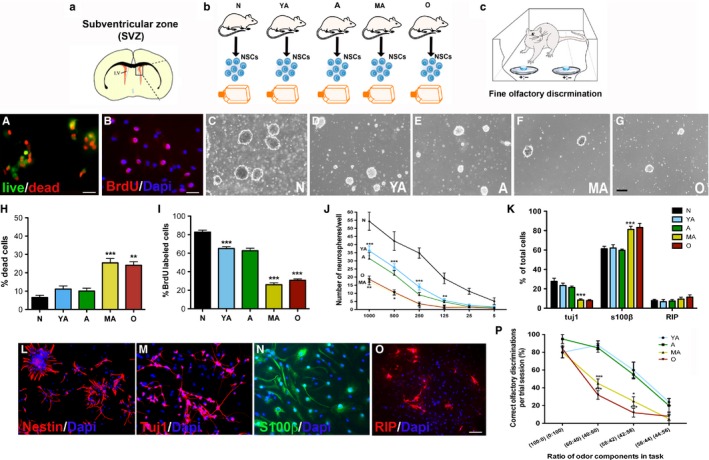
Characterization of NSPC survival and regenerative function across age. Cultured SVZ NSPCs from SVZs newborn (N), young adult (YA), adult (A), middle‐aged (MA), and old (O) rats were studied *in vitro* (a, b). Analysis via live–dead (A) and BrdU (B) assays are shown in H and I (H; *P* < 0.001, A vs. MA and *P* < 0.01 A vs. O: I; *P* < 0.001, N vs. YA, A vs. MA, and A vs. O). C–G depicts the capacity of the NSPCs to generate neurospheres with age, and results from a serial dilution assay are in J (*P* < 0.05, A vs. MA). Assessment of NSPC (L, nestin^+^) differentiation into Tuj1^+^ neurons (M), S100β^+^ astrocytes (N), and RIP ^+^ oligodendrocytes (O) is in K (*P* < 0.001, A vs. MA). The graph in P shows results from a fine olfactory discrimination task conducted on the aging rats (c) (*P < 0.05, **P < 0.01, ***P < 0.001; one‐way ANOVA with Tukey's *post hoc* in G‐I; two‐way RM‐ANOVA with Tukey's *post hoc* in J). Scale Bar: A‐B, 20 μm; C‐G, 200 μm; L, 50 μm; M‐O, 30 μm.

SVZ NSPCs isolated from five groups of rats, namely postnatal day 0 (newborn or N), 2 months (young adult or YA), 9 months (adult or A), 15 months (middle‐aged or MA), and 24 months (old or O), were analyzed *in vitro* (Fig. 1a,b). More specifically, NSPC survival (live–dead assay, Fig. 1A) and core NSPC properties of proliferation (5‐bromo‐2‐deoxyuridine (BrdU, Fig. 1B) and neurosphere assays, Fig. 1C‐G) and differentiation (immunohistochemistry, Fig. 1L‐O) were investigated. Both the live–dead and BrdU assays showed a progressive decline in NSPC survival and proliferation with increasing age (Fig. 1H, I). However, it was also observed that these NSPC properties exhibited a distinctive temporal pattern of age‐related decline across the age‐groups, with a strong decrement occurring in the MA (15 months) group of cells (survival: *P* < 0.001, *F*_4,10_ = 23.57, one‐way ANOVA; proliferation: *P* < 0.001, *F*_4,15_ = 133.7, one‐way ANOVA). Additionally, with respect to proliferation, a significant reduction (*P* < 0.001) was also noted in cells obtained from YA animals.

The proliferation of the SVZ NSPCs was further assessed through neurosphere assays. As depicted in Fig. 1C‐G, the ability of NSPCs to form neurospheres progressively decreased with age, supporting the notion that the proliferative potential of these age‐groups of cells was lower. However, given that NSPCs are truly a heterogenous mix of stem and progenitor cells with different proliferative capacities, we used a serial dilution assay, to allow for the differential analysis of stem and progenitor populations (Louis *et al*., 2008; Stoll *et al*., 2011). In this assay, it was noted that NSPCs from N animals produced a significantly greater number of neurospheres at every dilution tested compared to the adult age‐groups of cells (Fig. 1J). Among the adult age‐groups, significant differences were noted between A and MA cells with no differences observed between YA and A or MA and O cells (*P* < 0.01, two‐way ANOVA, Bonferonni's post‐test; *P* < 0.001, linear regression analysis). These data indicated that there were fewer stem‐like cells (with greater self‐renewal and proliferative potential) in the MA and O cultures compared to cultures from younger animals. In addition, we also performed a neural colony‐forming cell (NCFC) assay (Fig. S1A) (Louis *et al*., 2008). We adapted this assay (originally performed on embryonic cells) to the aging NSPCs and determined that not only the total number of spheres produced, but also the number of small (<0.5 mm, low proliferative potential) and large (0.5–1 mm, high proliferative potential), and stem‐like spheres (>1 mm, highest proliferative rate) significantly declined with advancing age, with a significant reduction observed in the MA group of cells (Fig. S1B‐E). These data altogether suggest that the proliferative capacity of both neural stem and progenitor populations was substantially compromised during aging, particularly in the context of the 9‐ to 15‐month MA time period.

The differentiation potential of the different age‐groups of NSPCs was also ascertained using immunohistochemistry. Here, it was observed that cells from all age‐groups expressed the immature NSPC marker nestin (Fig. 1L) under proliferative conditions, and upon mitogen withdrawal to induce differentiation showed the ability to generate neurons (Tuj1^+^, Fig. 1M), astrocytes (S100β^+^, Fig. 1N), and oligodendrocytes (RIP^+^, Fig. 1O), confirming their multipotency. However, further quantitative analysis revealed a pattern of age‐related vulnerability, similar to that observed in the proliferation and viability data, with a significant switch in NSPC phenotypic fate observed at the MA (15 months) stage (Fig. 1K). More specifically, while the number of RIP^+^ oligodendrocytes remained fairly constant, Tuj1^+^ neurons significantly declined (Fig. 1K, *P* < 0.001, *F*_4,58_ = 18.95, one‐way ANOVA) and the number of S100β^+^ astrocytes increased at the 15‐month MA time‐point (Fig. 1K, *P* < 0.001, *F*_4,58_ = 11.68, one‐way ANOVA).

To more carefully analyze the 9‐ to 15‐month period of increased NSPC vulnerability, we examined cells from two more groups of rats, aged 11 and 13 months. Analysis of the cells via the live–dead and BrdU assays revealed that a significant drop in survival (Fig. S2A, *P* < 0.05, *F*_6,14_ = 7.2, one‐way ANOVA) and proliferation (Fig. S2B, *P* < 0.05, *F*_6,15_ = 21.9, one‐way ANOVA) in fact occurred between 13 and 15 months. When NSPCs, from older animals aged 20 and 26 months, were assessed, no significant changes in either survival or proliferation were noted compared to the MA/O groups. These data establish a notable decline in NSC function during aging and identify a specific time window of enhanced cellular vulnerability, at 13–15 months, when there is a heightened reduction in NSPC function.

Moreover, we subjected the five primary age‐groups of animals to behavioral testing via a fine olfactory discrimination task, which is an established measure of the SVZ NSC function and neurogenesis *in vivo* (Fig. 1c; Enwere *et al*. (2004)). These results correlated with the cellular data from the *in vitro* analysis and indicated that MA and O animals were significantly worse (reflected by lower scores on the *Y*‐axis) than the young animals in discriminating almost equal ratios of [+]/good tasting coconut (COC) and [–]/bad tasting mixture of almond and denatonium benzoate (ALM) (Fig. 1P; *P* < 0.001, two‐way RM‐ANOVA). More specifically, all age‐groups of rats were equally good at discriminating between pure solutions of COC [+] and ALM [‐] indicating that their discrete odor discrimination capacity was not impaired. However, when their fine odor discrimination ability was tested by exposing them to blended solutions containing similar amounts of COC and ALM (ratios of 60:40, 58:42 or 56:44), the performance of the MA rats declined sharply in comparison with the adult (9 months old) rats.

### The pattern of age‐related decline in NSPC survival and regenerative function correlates with reduced Nrf2 expression *in vitro*

**Figure 2:**
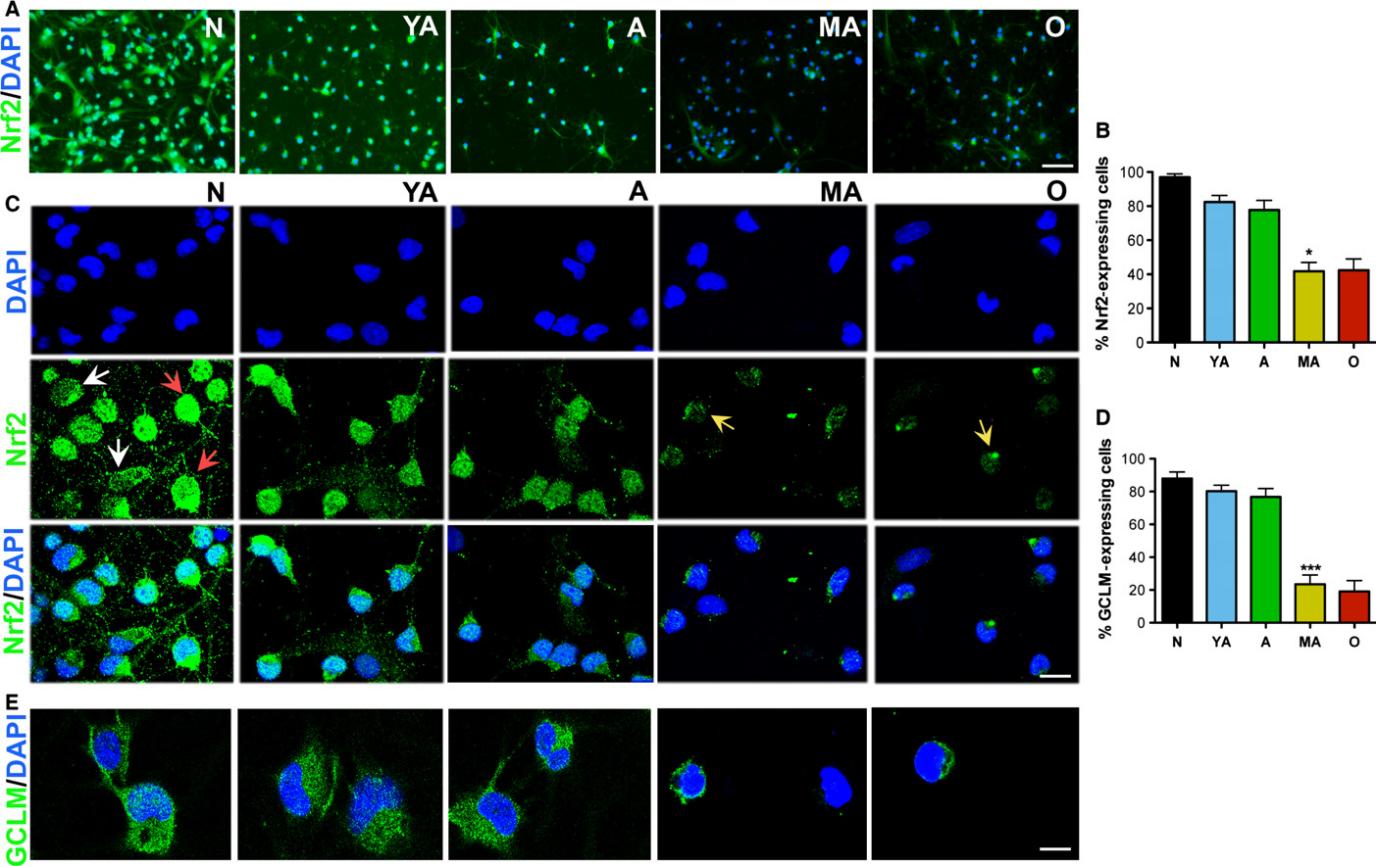
Cultured NSPCs show reduced Nrf2 expression/activity with advancing age. Low magnification images of Nrf2 expression across age are shown in (A), with quantification in (B) (*P* < 0.05, A vs. MA,). High magnification confocal images of Nrf2 expression are shown in (C) [Nrf2 expression in the cytoplasm (white arrows), and/or nucleus (red arrows) in younger cells, low or no Nrf2 expression in MA and O cells (yellow arrows)]. Analysis of expression of the Nrf2 target gene, GCLM, is in E and D (*P* < 0.01, A vs. MA). [*P < 0.05, ***P < 0.001, one‐way ANOVA,* post hoc* Tukey's test]. Scale Bar: A, 50 μm; C, 15 μm; E, 10 μm.

Our prior studies have demonstrated the increased expression of antioxidants as an essential feature of NSPCs, which determines their survival, proliferation, differentiation, and therapeutic function (Madhavan *et al*., 2006, 2007). Therefore, we examined Nrf2 expression in the 5 age‐groups of SVZ NSPCs, using immunocytochemistry (Fig. 2). Qualitatively, the NSPC age‐groups showed decreased Nrf2 expression with age (Fig. 2A). More specifically, confocal imaging revealed that N, YA, and A cells showed high Nrf2 expression, noted in the cytoplasm and/or nucleus, whereas no or low Nrf2 expression was observed in the MA and O cells (Fig. 2C). Additional quantitative analysis indicated an age‐related reduction in the percentage of cultured NSPCs expressing Nrf2, which closely mirrored the pattern of decrease in NSPC survival and proliferation with age (Fig. 2B). Again, the 15‐month‐old cells were the first to show a significant loss of Nrf2 across the age‐groups (*P* < 0.05, compared to A, *F*_4,10_ = 20.23, one‐way ANOVA). Furthermore, the fraction of Nrf2‐expressing cells in NSPC cultures grown at 3% O_2_ (low oxygen) was also quantified, given that Nrf2 is redox sensitive. In this scenario, a similar pattern of decline in number of Nrf2‐expressing cells, as observed under ambient laboratory conditions (21% O_2_), was observed (Fig. S1I, *P* < 0.01, compared to A, *F*_4,10_ = 44.7, one‐way ANOVA). In addition to Nrf2 expression, NSPC survival and proliferation under low O_2_ (Fig. S1F‐H) also showed a declining trend across age, with a significant drop occurring in middle age, comparable to what was observed in NSPCs grown under ambient laboratory conditions (Fig. 1), thus highlighting the robustness of this age‐related phenomenon. Also, SVZ Nrf2 expression in 11‐, 13‐, 20‐, and 26‐month‐old animals was studied (Fig. S2C). In support of the critical MA period defined previously with regard to NSPC survival and function, these data revealed that the decline in Nrf2 expression also occurred between 13 and 15 months of age after which no alterations were seen until 26 months.

To assess Nrf2 activity, we immunocytochemically analyzed the expression of three classical Nrf2 target genes, glutamate–cysteine ligase modifier subunit (GCLM), NADPH: quinone oxidoreductase (NQO1), and heme oxygenase 1 (HO1), in the NSPCs. As shown in Fig. 2D and E, N, YA, and A NSPCs showed high GCLM expression in a large percentage of cells, compared to MA and O NSPCs. However, NQO1 and HO1 expression was hardly detected in any of the aging NSPC groups, although occasional (~5% of cells) NQO1 expression was noted in newborn NSPCs (data not shown).

### Correlation of declining Nrf2 expression with aging NSPC function *in vivo*

**Figure 3:**
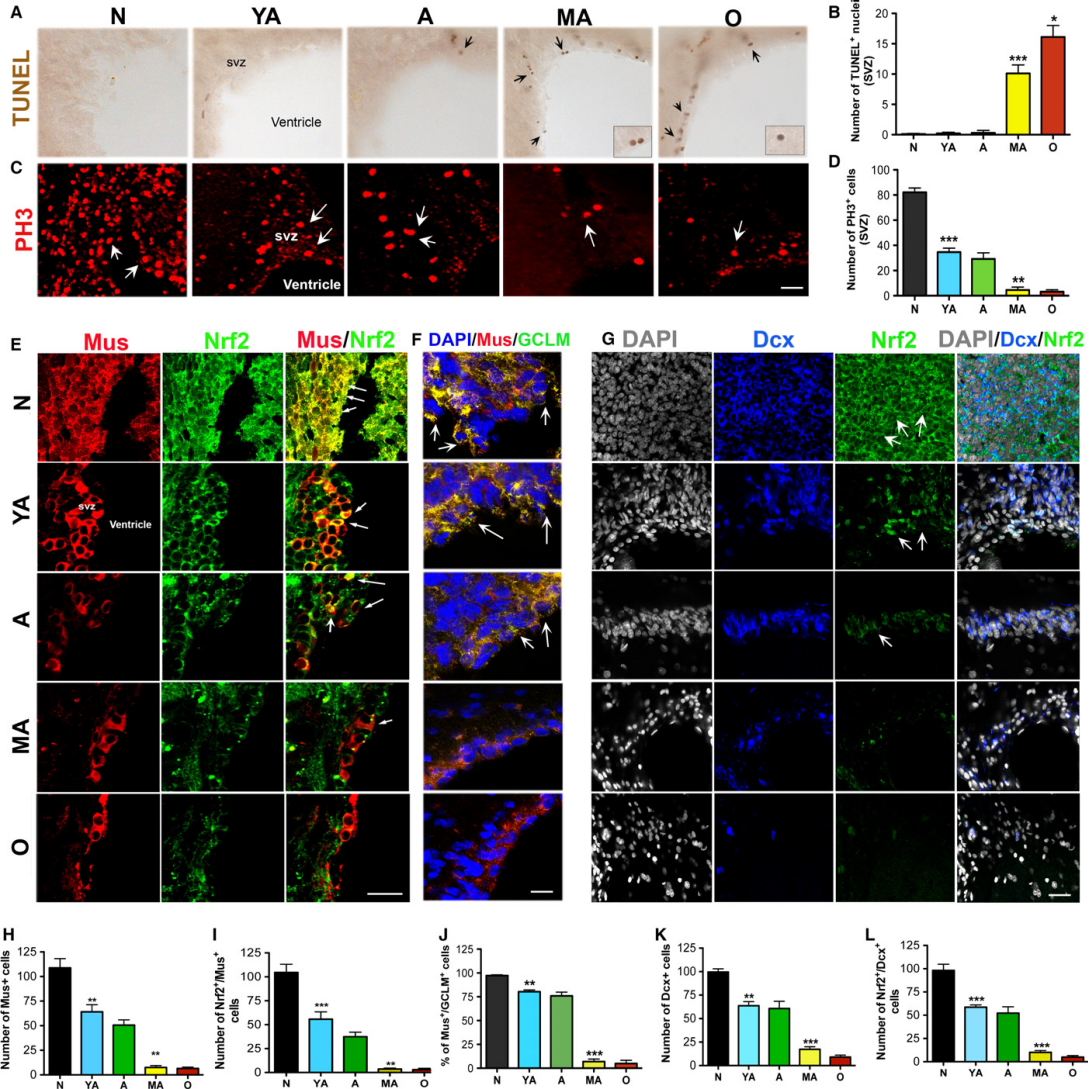
*In vivo* characterization of NSPC survival, regeneration, and Nrf2 expression/activity. Results from a TUNEL staining (for cell death) are in A and B, and PH3 staining (for proliferation) in the SVZ is in C and D. Assessment of SVZ Mus^+^ NSPCs and Dcx^+^ neuroblasts are in E, H, G, and K (*P* < 0.01 N vs. YA,* P* < 0.001, A vs. MA). Analysis of Nrf2 and GCLM expression in the Mus^+^ cells appears in E, F, I (*P* < 0.001, N vs. YA and *P* < 0.01, A vs. MA), and J (*P* < 0.01, N vs. YA; and *P* < 0.001, A vs. MA). Analysis of Nrf2 in Dcx^+^ NSPCs is in G and L (*P* < 0.001, N vs. YA, A vs. MA). White arrows point to examples of positively immunostained cells. [*P < 0.05, **P < 0.01, ***P < 0.001, one‐way ANOVA,* post hoc* Tukey's test]. Scale bars: A, 50 μm; C, E, 25 μm; F, G, 20 μm.

Nrf2 expression was also studied *in vivo* in the SVZ niche (Fig. 3). First, in support of the *in vitro* data, greater number of TUNEL^+^ (marks apoptosis) nuclei were observed in the SVZs of MA and O rats, indicating greater ongoing cell death in this region (Fig. 3A, B). Secondly, the number of NSPCs expressing phosphohistone 3 (PH3, a mitotic marker), as well as Musashi 1 (Mus, marks a large fraction of SVZ stem and progenitors), declined significantly with age with a notable reduction observed at MA (Fig. 3C, D, *P* < 0.001, *F*_4,10_ = 65.2, one‐way ANOVA; Fig. 3E, H; *P* < 0.001, *F*_4,10_ = 52.4, one‐way ANOVA). Thirdly, correlating with these data, Nrf2 expression in the SVZ, as well as the number of SVZ cells expressing both Musashi1 and Nrf2, was found to significantly decline with advancing age, with a noteworthy reduction seen at middle age (Fig. 3E, I). Lastly, similar to what was observed *in vitro*, quantitative analysis showed that the reduction in PH3, Mus, and Nrf2 expression, during middle age, occurred precisely between 13 and 15 months after which it remained relatively steady (Sup Fig. 2D‐F). It was determined that Mus^+^ SVZ cells in N, YA, and A animals had high levels of the Nrf2 target gene GCLM, indicating activation of the Nrf2 pathway (yellow cells in Fig. 3F, J) compared to MA and O animals, but no significant expression of NQO1 or HO1 was seen.

SVZ neurogenesis was also evaluated by examining the expression of doublecortin (Dcx, a marker of newly born neuroblasts). As expected, the number of Dcx^+^ cells in the dorsolateral SVZ decreased with increasing age (Fig. 3G), with again a significant reduction seen between 9 and 15 months (Fig. 3K). The number of Nrf2‐expressing Dcx^+^ neuroblasts also decreased in similar manner, as shown in Fig. 3G and L.

### Alteration of Nrf2 expression significantly impacts NSPC survival and function

**Figure 4:**
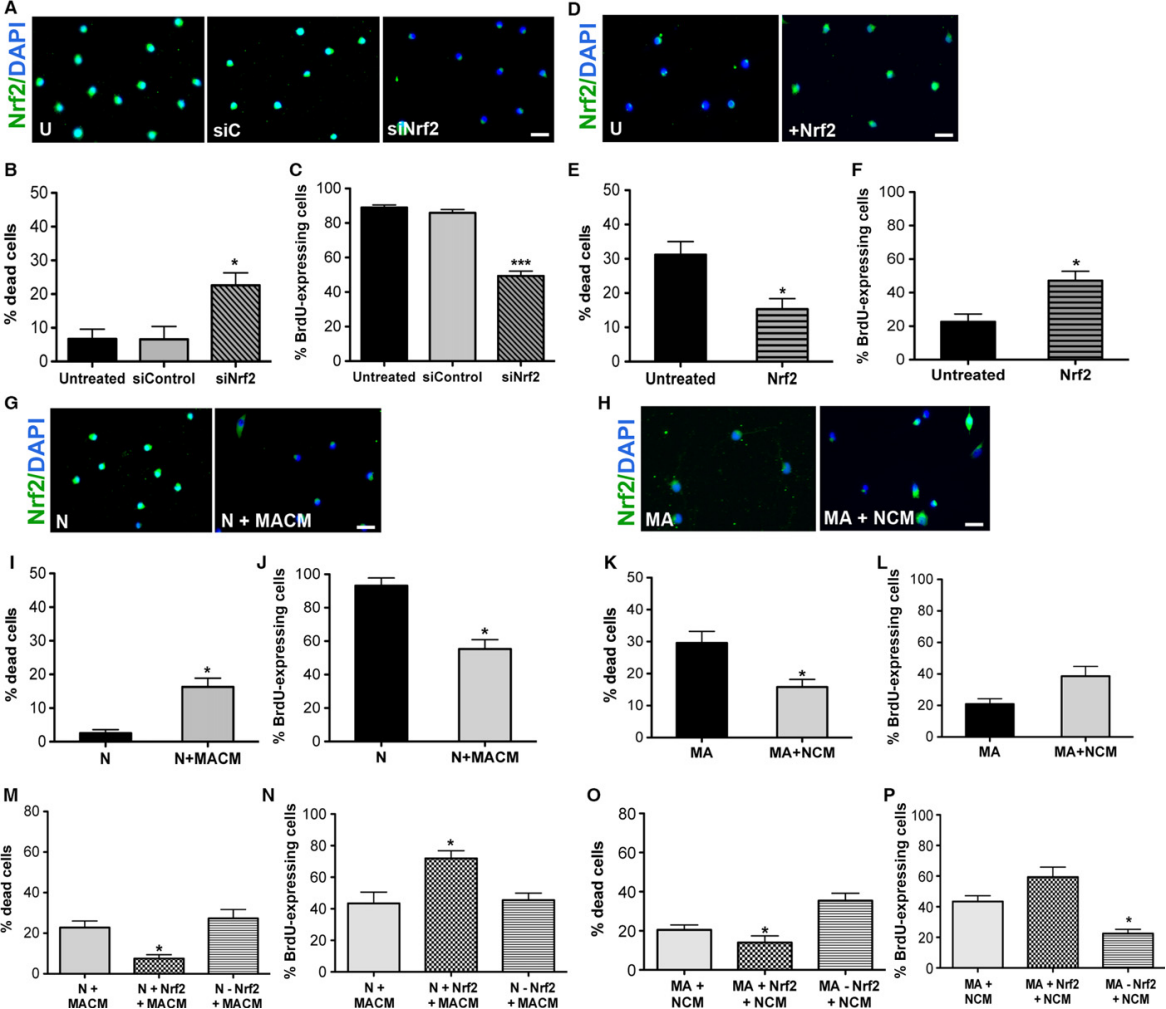
Effect of altered Nrf2 expression on NSPC survival and function. Panel A shows examples of Nrf2 expression with or without knockdown of Nrf2 in the N cells. Graphs in B and C show results from live–dead and BrdU assays conducted on untreated (U), control siRNA (siC), and Nrf2 siRNA (siNrf2)‐treated N cells. Panel D shows Nrf2 expression with and without Nrf2 overexpression in MA cells, with parallel results from live–dead and BrdU assays conducted on untreated MA cells compared to those transfected with Nrf2 in E and F. I and J convey results from similar experiments in which N cells were treated with conditioned medium (CM) from MA cells (MACM), with corresponding Nrf2 expression shown in panel G. The effect of CM from newborn NSPC cultures (NCM) on MA cells is in K and L, with corresponding changes in Nrf2 expression in panel H. The effects of NCM or MACM, on the MA and N cells, respectively, with Nrf2 knockdown or overexpression are shown in M‐P [*P < 0.05, ***P < 0.001; one‐way ANOVA with *post hoc* Tukey's test for B, C, and M–P, unpaired *t*‐test for E, F, I‐L]. Scale bar: 25 μm.

Given the data on its significantly declining expression in SVZ NSPCs, we further assessed Nrf2 through *in vitro* knockdown (RNA interference) and overexpression assays. Using targeted short interfering RNAs, Nrf2 was silenced in newborn (N) cells after which the cells were analyzed via live–dead and BrdU assays (Fig. 4A shows Nrf2 expression with and without knockdown). These data indicate that Nrf2 knockdown renders the N cells similar to ‘older’ cells, with low survival (Fig. 4B, *P* < 0.05, *F*_2,6_ = 6.9, one‐way ANOVA) and low proliferative rates (Fig. 4C, *P* < 0.001, *F*_2,6_ = 108.3, one‐way ANOVA), as compared to untreated (U) and silencing controls (siC). On the other hand, when MA NSPCs were transfected with Nrf2 (Fig. 4D shows Nrf2 expression with and without overexpression), their survival (Fig. 4E, *P* < 0.05, unpaired *t*‐test, *t* = 3.2, df = 3) and proliferation (Fig. 4F, *P* < 0.05, unpaired *t*‐test, *t* = 3.4, df = 3) was significantly improved, making them akin to ‘younger’ cells. These data indicated that simply altering intrinsic Nrf2 expression was sufficient to significantly change NSPC survival and function, supporting an important regulatory role for this factor in NSPC function during aging.

To study how extrinsic factors, in the environment of the aging NSPCs, may influence NSPC function and Nrf2 expression, we treated the N NSPCs with conditioned medium (CM) obtained from MA NSPC cultures (MACM). Alternatively, MA NSPCs were also exposed to CM from N cultures (NCM). As shown, when the N cells were assessed after 1 week of exposure to MACM, it was observed that their survival (Fig. 4I, *P* < 0.05, unpaired *t*‐test, *t* = 3.1, df = 3) and proliferation (Fig. 4J, *P* < 0.05, unpaired *t*‐test, *t* = 5.2, df = 3) had been significantly reduced under these conditions. On the other hand, the survival (Fig. 4K, *P* < 0.05, unpaired *t*‐test, *t* = 3.1, df = 3) of MA cells treated with NCM had substantially improved, while proliferation although increased was not statistically significant (Fig. 4L, *P* > 0.05, unpaired *t*‐test, *t* = 5.4, df = 3). Parallel examination of NSPC Nrf2 expression indicated that the reduction in survival and proliferation in the MACM‐treated N cells was associated with reduced Nrf2 in the NSPCs (Fig. 4G, 68.5 ± 4.5 Nrf2‐expressing cells in N + MACM cultures compared to 98.4 ± 3.6 in N cultures). Similarly, the enhanced survival and proliferation of the NCM‐treated MA NSPCs was associated with visibly increased Nrf2 expression in the NSPCs (Fig. 4H, 56.8 ± 4.2 Nrf2‐expressing cells in MA + NCM cultures compared to 38.4 ± 4.9 in MA cultures).

Given the changes in Nrf2 expression observed during the above CM experiments, we further investigated how CM mediated cell‐extrinsic regulation of rat NSPC function is affected by Nrf2 loss or gain of function. When N cells were silenced for Nrf2 and treated with MACM, no significant changes in NSPC survival or proliferation were observed, compared to the MACM‐only treatment condition, suggesting that reduced Nrf2 may partly be involved in mediating the MACM effects (Fig. 4M, N). Nrf2 overexpression on the other hand was able to counter the effects of MACM. Treatment of MA cells with NCM after Nrf2 overexpression exaggerated the positive effects of NCM on the survival and proliferation on the NSPCs (Fig. 4O, P). In contrast, Nrf2 knockdown interfered with NCM effects, suggesting that NCM probably acts via Nrf2 in exerting its cell‐extrinsic influences in promoting a ‘young state’ in the MA cells. These data implicate a role for diffusible factors from newborn and MA NSPCs in mediating Nrf2 expression and NSPC survival/proliferative function.

### SVZ NSPCs from Nrf2 knockout mice show deficits in survival, proliferation, and neuronal differentiation

**Figure 5:**
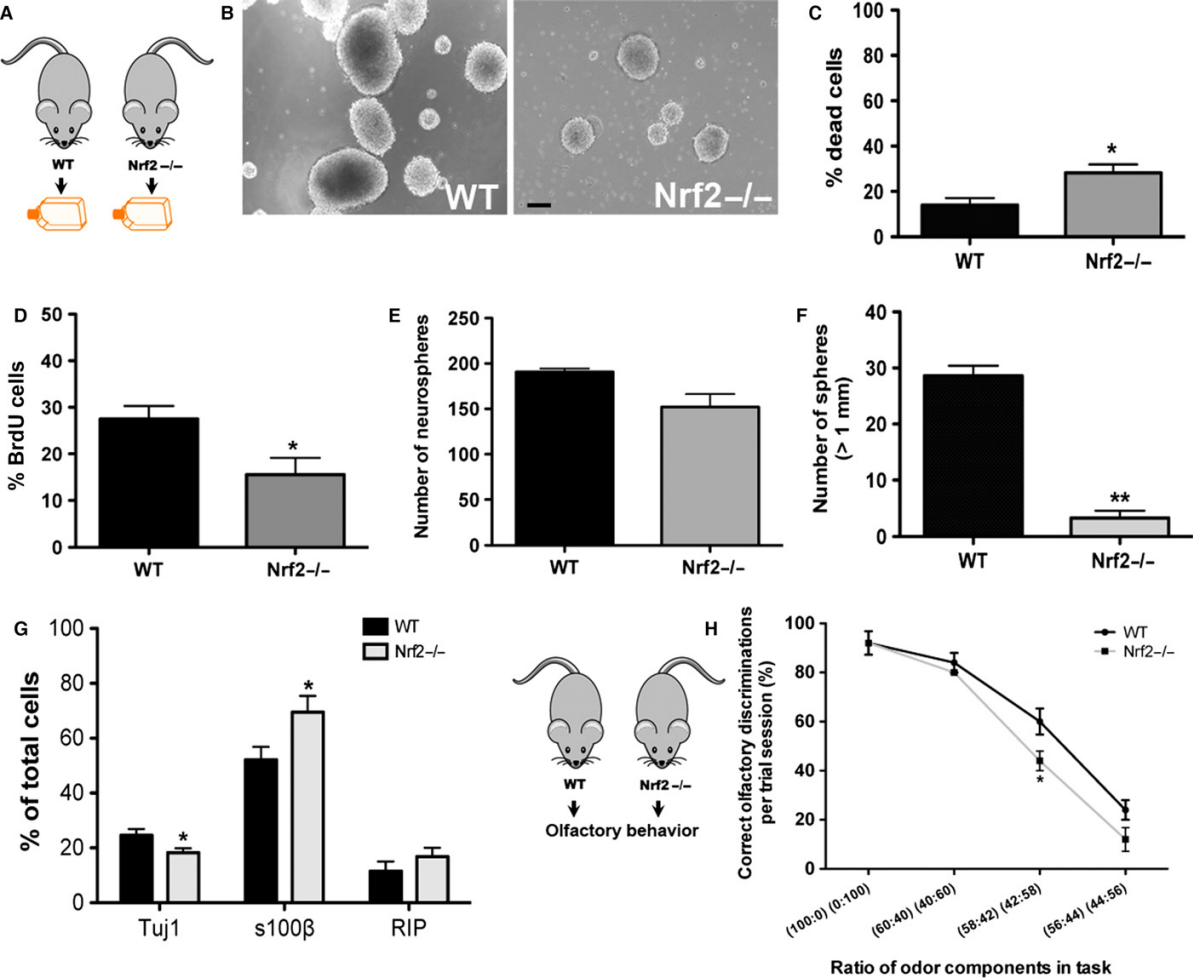
NSPCs from Nrf2 knockout animals show reduced survival and proliferation. *In vitro,* SVZ NSPCs from newborn Nrf2 knockout (Nrf2−/−) and WT mice were isolated (A) and subjected to live–dead (C), BrdU (D), and neurosphere (B, E, F). The differentiation of the Nrf2−/− and WT cells was also assessed (G). Behavioral analysis of young adult WT and Nrf2−/− animals via a fine olfactory discrimination test is in H [*P < 0.05, **P < 0.01, unpaired *t*‐tests in C‐F, one‐way ANOVA with *post hoc* Tukey's test in G]. Scale bar: B, 300 μm.

To further study the role of Nrf2, NSPCs isolated from newborn Nrf2 knockout mice (Nrf2−/−), and corresponding wild‐type controls, were assessed *in vitro* (Fig. 5A). When NSPCs obtained from the SVZs of Nrf2−/− and WT mice were compared via a live–dead assay, it was determined that the viability of the knockout cells was significantly lower (Fig. 5C, *P* < 0.05, unpaired *t*‐test *t* = 2.9, df = 9). With respect to proliferation, BrdU labeling indicated that there were significantly less actively dividing NSPCs in Nrf2−/− cultures (Fig. 5D, *P* < 0.05, unpaired *t*‐test, *t* = 2.6, df = 17). In the neurosphere assay, it was determined that although the total number of spheres generated was not significantly different (Fig. 5E), the fraction of neurospheres ≥1 mm in size was substantially reduced denoting that the regenerative function of stem‐like cells in the cultures had been compromised (Fig. 5B, F; *P* < 0.01, unpaired *t*‐test, *t* = 11.3, df = 3). Moreover, when the differentiation of the NSPCs was examined, the KO cells generated significantly lower numbers of neurons, but higher numbers of astrocytes, compared to the WT cells (Fig. 5G). Also, when the fine olfactory discrimination ability of the mice at the YA stage was examined, the KO animals exhibited a reduced capacity to discriminate between very similar ratios of COC and ALM (58:42 or 56:44) compared to the age‐matched YA WT mice (Fig. 5H).

**Figure 6:**
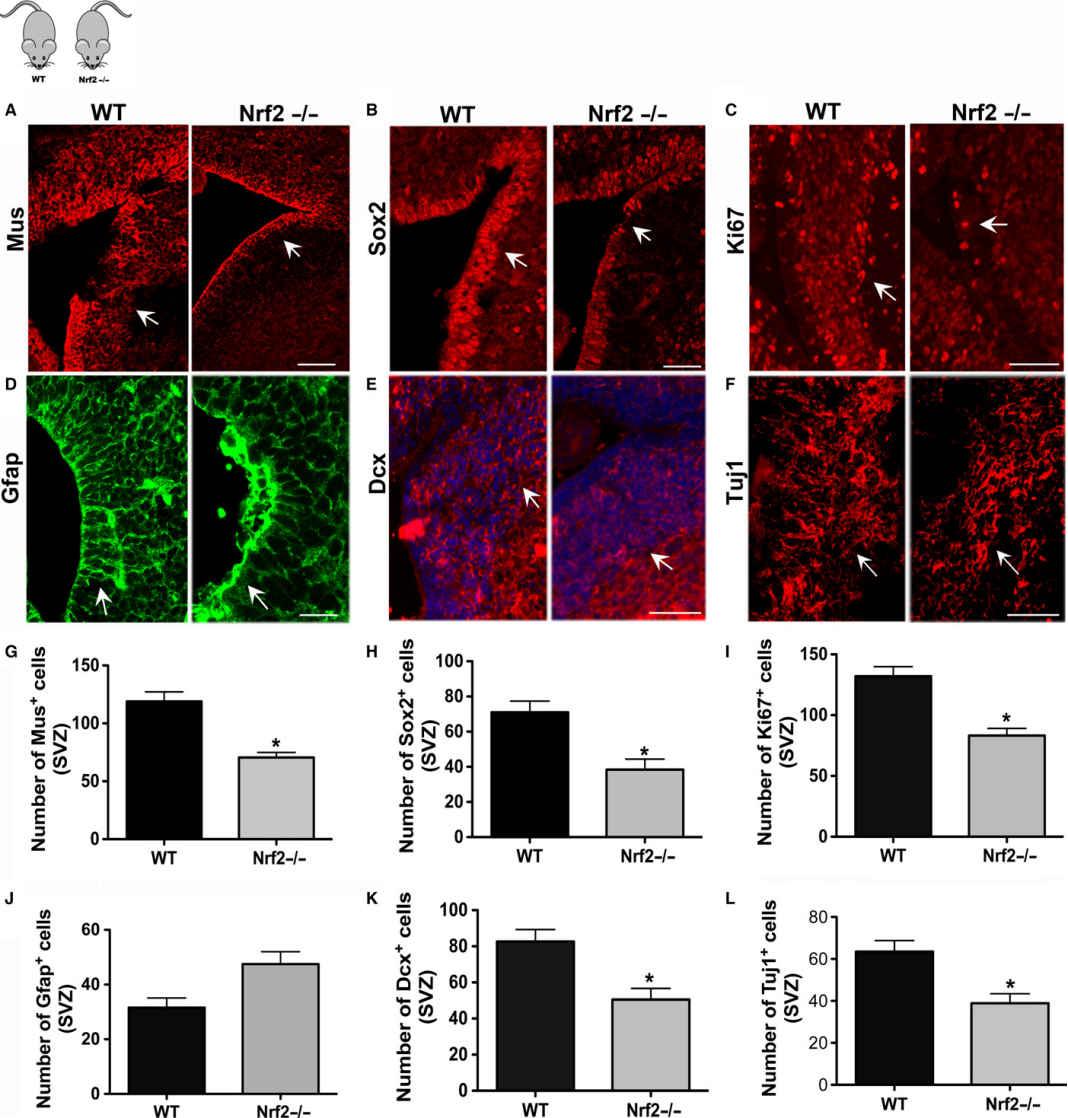
*In vivo* analysis of SVZ NSPCs in Nrf2 knockout mice. Immunohistochemical analysis on the SVZs of newborn Nrf2−/− and WT mice using antibodies targeting Musashi1 (A, G), Sox2 (B, H), Dcx (E, K), Tuj1 (F, L), GFAP (D, J), and Ki67 (C, I) is shown. [*P < 0.05, unpaired *t*‐tests]. Scale Bars A‐E, 100 μm.

We also examined the effects of Nrf2 knockout on SVZ NSPCs *in vivo,* in the newborn animals, by studying the expression of antigens that identify SVZ stem and progenitor cells, and their progeny. Specifically, we examined cells positive for Musashi1 and Sox2 (expressed in type B and C NSPCs), doublecortin (expressed in type A NSPCs, an indicator of neurogenesis), Tuj1 (neuronal marker), and GFAP (expressed by type B NSPCs, glial marker). We also studied cellular proliferation in the SVZ using Ki67 as a marker. These data indicated that Musashi1 (Fig. 6A, G; *P* < 0.05, unpaired *t*‐test, *t* = 5.1, df = 3) and Sox2 (Fig. 6B, H; *P* < 0.05, unpaired *t*‐test, *t* = 13.7, df = 3) expressing NSPCs were significantly reduced in the Nrf2−/− animals compared to WT controls. In addition, Dcx^+^ neuronal progenitors and Tuj1^+^ neurons (Fig. 6E, K; *P* < 0.05, unpaired *t*‐test, *t* = 3.5, df = 3; Fig. 6F,L; *P* < 0.05, unpaired *t*‐test, *t* = 3.5, df = 3) were significantly lower, whereas GFAP^+^ cells were present in greater numbers (although not significantly, Fig. 6D, J). These data supported the *in vitro* data in Fig. 5 and indicated that neuronal and glial differentiation had been altered in the Nrf2−/− animals. Finally, the number of Ki67^+^ cells was determined to be significantly lower in the SVZ of the Nrf2−/− mice, indicating that the proliferative potential of the NSPCs had also been dampened (Fig. 6C, I; *P* < 0.01, unpaired *t*‐test, *t* = 4.9, df = 3).

## Discussion

In essence, our study makes the following novel contributions: (i) the specific delineation of a critical middle‐age period of vulnerability during aging when NSPC survival and function is significantly compromised, (ii) the determination of an impairment in fine olfactory discrimination ability in the aging rats, during the critical period, underlining the functional relevance of the decline in NSPC function, and (iii) the demonstration of a reduced expression of Nrf2, in the NSPCs, as an important mechanism mediating the age‐related NSPC deterioration. These results, for the first time, identify Nrf2 as an important intrinsic factor contributing to the decline in NSPC function in relation to a critical time during aging and have important implications toward understanding fundamental aspects of NSPC biology and potentially improving NSPC function with advancing age.

First, the present study provides a precise demarcation of the time at which NSPCs are compromised during the life course. While previous studies have described NSPC morphology and function at young and old endpoints of the age spectrum, the present study provides a characterization of how the NSPC population changes across the lifespan in multiple groups of animals at various stages of aging (Conover & Shook, 2011; Stoll *et al*., 2011). By systematically examining NSPC survival, proliferation/self‐renewal, and differentiation *in vitro* and *in vivo*, we demonstrate a specific time period during middle age, in particular 13–15 months, when NSPC survival and regeneration declines considerably. Critically, we also show that the specific temporal pattern of NSPC deterioration is associated with behavioral (functional) consequences, namely the impairment of fine olfactory discrimination abilities, in the aging rats.

In this context, previous reports have indicated that the number of proliferating cells in the mouse SVZ declines by mid‐age (10 or 12 months), with an additional reduction at 22 months (Luo *et al*., 2006; Bouab *et al*., 2011). However, no alterations in NSPC survival were found, and it was proposed that the decrease in NSPC number in the mice occurred due to quiescence‐associated changes (cessation of stem cell division) as opposed to changes in NSPC viability. Our data do not fully support this hypothesis as they show a sudden and dramatic drop in both NSPC survival and proliferation, especially between 13 and 15 months of age, after which the rate of NSPC survival and regeneration remains relatively constant until a later age of 26 months. Additionally, our studies determine a change in the phenotypic fate of the NSPCs by 15 months, at which point the NSPC's potential to generate astrocytes increases, but neuronal differentiation decreases. These data align with the studies mentioned above, which report greater numbers of astrocytes in the SVZs of old animals, and further suggest that the upsurge in the SVZ astrocytic population may in fact be occurring between 13 and 15 months of age (Luo *et al*., 2006). Furthermore, our study also connects the observed temporal pattern of decline in NSPC function with behavioral impairment in the aging rats, which is new. Regardless of the apparent discrepancies between the studies, taken together, they support the idea that there is a significant decline in NSPC function by middle age. Because of the systematic examination across age in the present study, we were able to show that a significant reduction in NSPC survival and function in fact occurs quite swiftly at 13–15 months in the F344 rat. This rapid decline in middle age highlights the sensitivity and the finely regulated nature of the NSPC aging process. Moreover, the delineation of this particular critical period also has implications in terms of the identification of promising time windows during aging when therapeutic interventions may be most successfully applied.

The second important contribution of this work lies in the discovery of Nrf2 as an important intrinsic factor controlling NSPC function during aging. In particular, our results indicate that the specific temporal pattern of NSPC decline during middle age correlates with reduced expression of Nrf2 and its target gene GCLM in NSPCs. Additionally, decreasing or increasing Nrf2 expression was sufficient to significantly suppress or rejuvenate the survival and regenerative capacity of newborn or middle‐aged rat NSPCs, respectively. Furthermore, newborn SVZ NSPCs in Nrf2 knockout mice exhibited substantially compromised survival, proliferation, neuronal differentiation, and fine olfactory discrimination ability compared to cells from wild‐type mice. Previous research has provided clues on plausible processes that may be responsible for the NSPC declination with age by investigating the influence of growth factor signaling on neurogenesis in aged animals, or using models where aspects of NSPC aging are mimicked (Tropepe *et al*., 1997; Enwere *et al*., 2004; Molofsky *et al*., 2006; Nishino *et al*., 2008). In contrast, the current work directly identifies Nrf2 as a key mediator of the compromise in NSPC function during the sequence of ‘normal’ aging.

Nrf2 in fact seems well poised to significantly influence NSPC survival and function. Classically, Nrf2, when activated, increases the expression of multiple cell survival mechanisms such as antioxidant, anti‐inflammatory, and other cytoprotective pathways through the binding of antioxidant response elements (AREs) in the promoter region of its target genes (Lee *et al*., 2005; Bryan *et al*., 2013). By doing so, Nrf2 can control a range of cellular stressors (including reactive oxygen species or ROS), and ultimately promote a reduced intracellular environment which is being increasingly recognized as critical to NSPC survival and fate (Ramalho‐Santos *et al*., 2002; Madhavan *et al*., 2006, 2007; Noble, 2006; Rafalski & Brunet, 2011). Specifically, it has been reported that a highly reduced intracellular redox state (diminished ROS) promotes proliferation and survival, whereas a highly oxidized state (excessive ROS) results in greater differentiation and apoptosis (Smith *et al*., 2000). In addition to its classical function in regulating antioxidant response, recent studies also reveal that Nrf2 controls a global cellular protective response to promote cell survival by regulating the expression of many classes of genes beyond antioxidant genes and also partakes in other cellular functions. Among its many roles, Nrf2 has been linked to cell growth, self‐renewal, differentiation, proliferation, and increased lifespan, which are clearly relevant to NSPC function (Wakabayashi *et al*., 2010; Zhu *et al*., 2013). Further, Nrf2 can also control mitochondrial and trophic factor activities, which crucially support NSPC survival and function (Bryan *et al*., 2013; Holmstrom *et al*., 2013; Wiesner *et al*., 2013). In this context, our results suggest the potential involvement of GCLM in mediating the observed Nrf2‐based effects. However, the exact molecular pathways involved will need to be investigated.

It is realized that intrinsic molecular changes within NSPCs intricately connect to alterations occurring in the extrinsic tissue environment during aging to regulate NSPC function and fate. In this regard, recent studies in parabiotic pairings between old and young mice suggest that aged endogenous NSCs can be somewhat revived when provided extrinsic factors from younger environments (Villeda *et al*., 2014). The results from our *in vitro* analysis are consistent with these observations and indicate that the regeneration and survival of newborn and middle‐aged NSPCs can be altered by exposure to conditioned medium from the other cell type. Interestingly, such conditioned medium induced improvement or degradation of NSPC function appeared to affect and in part be mediated via Nrf2. Thus, it will be important to investigate the relationship between factors such as increased oxidative stress, inflammation, and reduced growth factors (which are natural stressors in the aging tissue environment), Nrf2 expression, and NSPC function.

In conclusion, our study identifies a critical time during middle age when there is a notable reduction in NSPC survival and regeneration, and implicates the reduced expression of Nrf2 as key in mediating this phenomenon. The recognition of a precise time period during which Nrf2 expression is altered and impacts NSPC function offers the opportunity to understand fundamental aspects of NSPC dynamics with age. Importantly, this should lay the foundation for the development of targeted NSPC‐based strategies to promote healthy aging and to treat age‐related neural disorders.

## Experimental procedures (Detailed descriptions provided in the Supplementary section)

### Animals

Newborn postnatal day 0 pups (termed newborn or N) and adult male Fisher 344 rats of various age‐groups, mainly 2 (young adult or YA), 9 (Adult or A), 15 (middle‐aged or MA), and 24 (Old or O) months of age, were used (NIH‐NIA, Bethesda, MD; Harlan Laboratories, Indianapolis, IN, USA). Corresponding approximate ages in human years are mentioned in Table 1 below. Additionally, 11‐, 13‐, 20‐, and 26‐month‐old rats were also used in some experiments. All animals were housed at the Animal Care Facility at The University of Arizona and were kept on a reverse 12‐h light–dark cycle with food and water available *ad libitum*. Newborn (postnatal day 0) Nrf2^+/+^ (WT) and Nrf2^−/−^ (KO) C57BL/6 mice were also maintained at the University of Arizona animal care facility (Chan *et al*., 1996; Tao *et al*., 2013). All animals were treated according to the rules and regulations of NIH and Institutional Guidelines on the Care and Use of Animals. The University of Arizona Institutional Animal Care and Use Committee specifically approved all experimental procedures.

**Table 1:** Comparison of rat and human age

Age	N	YA	A	MA	O
Rat (Months)	0	2	9	15	24
Human (Years)	0	15	30	45	70

For isolating primary NSPCs, animals were sacrificed using sodium pentobarbital (60 mg kg^−1^), and brains microdissected to obtain SVZ tissues. For histology, animals were perfused with 4% paraformaldehyde (PFA), and brains extracted and sectioned coronally at 40 μm on a freezing sliding microtome or on a cryostat at 10 μm thickness.

### NSPC culture

SVZ NSPCs from newborn rats, Nrf2−/− and WT mice, and adult rats of various ages were cultured using established methods (Madhavan *et al*., 2009). All subsequent experiments were conducted on passage 1–4 NSPCs, with all assays involving at least *n* = 3 independent cultures of NSPCs, grown in parallel, and examined in triplicate for each age‐group.

### NSPC survival

NSPC viability was assessed using a live–dead cell assay kit (Life Technologies, Grand Island, NY, USA) as explained in the supplementary methods section.

### NSPC proliferation

#### BrdU assay

NSPCs were plated on poly‐D‐lysine‐/laminin‐coated glass coverslips in proliferation medium, treated with 10 μm BrdU (Sigma‐Aldrich, St. Louis, MO, USA) for 1–1/2 h, fixed in 4% PFA, and subsequently immunostained with antibodies targeting BrdU and counterstained with DAPI (4′,6′‐diamidino‐2‐phenylindole, dihydrochloride; Life Technologies). The number of BrdU‐ and DAPI‐labeled cells was counted in 5 fields per coverslip under a 20× lens.

#### Neurosphere assay

*Total sphere counts—*NSPCs were grown in proliferation medium as nonadherent cultures and allowed to form neurospheres. The number of neurospheres generated was counted in 15 fields per culture dish under a 20× lens (Zeiss Observer A1 microscope, AxioCam Mrc digital camera, Axiovision software; Carl Zeiss, Oberkochen, Germany).

#### Serial dilution assay

NSPCs were plated at increasing concentrations of 5, 25, 125, 250, 500, and 1000 cells per well in a 96‐well plate in proliferation medium. After 7 days in culture, the number of neurospheres/well formed under these various NSPC plating densities was counted per well under a 20× lens.

#### NCFC assay

The NCFC assay was carried out using the NeuroCult Rat NCFC Assay Kit (Stemcell Technologies, Vancouver, BC, Canada) as detailed in supplementary section).

### NSPC differentiation

NSPCs were plated onto poly‐D‐lysine‐/laminin‐coated glass coverslips in 24‐well plates and induced to differentiate (details in supplementary section). After 10 days in culture, 5 fields per coverslip were assessed and the percentage of DAPI‐stained cells expressing Tuj1 (neurons), S100β (astrocytes), or RIP (oligodendrocytes) were counted under a 20× lens.

### Immunocytochemistry

NSPCs plated on poly‐D‐lysine/laminin‐coated glass coverslips were immunostained following published protocols (Madhavan *et al*., 2009). Briefly, cells were washed and blocked with 1% BSA in 1× PBS containing 0.4% Triton X‐100 and 2% normal goat serum. After incubation overnight at 4°C with relevant primary antibodies (detailed description in supplementary methods), cells were treated with secondary antibodies (1:500) coupled to fluorochromes Alexa 488, 594, or 647 (Life Technologies‐Molecular Probes, Grand Island, NY, USA) and counterstained with DAPI.

### Cell counts

*In vitro*, the number of DAPI‐labeled NSPCs expressing Nrf2 (staining covering most of the nucleus and/or cytoplasm was considered positive) and GCLM (staining most of the cytoplasm) was counted in 5 fields per sample under a 20× lens (Zeiss Axioimager M2). *In vivo*, the number of SVZ cells expressing Nrf2, GCLM, Musashi, Dcx, and PH3 in the rats, and the number of SVZ cells expressing Musashi, Sox2, Ki67, Dcx, and GFAP in Nrf2−/− and WT mice were counted in 6 adjacent sections per animal, under a 63× lens of a confocal microscope (Leica SP5‐II with LAS software, Leica Microsystems, Buffalo Grove, IL). Counts were performed on DAPI‐counterstained sections with Z sectioning performed at 1‐ to 2‐μm intervals to identify individual cells and the colocalization of markers.

### siRNA and transfection assays

NSPCs from newborn (P0) or middle‐aged (15 months) rats were treated with siRNAs (Santa Cruz Biotechnology, Dallas, TX, USA) targeting Nrf2, or transfected with Nrf2, as outlined in the Supplementary section.

### Fine olfactory discrimination behavior

Protocols from Enwere *et al*., 2004 were followed, details of which are provided in the Supplementary section.

### Immunohistochemistry and TUNEL staining

Immunohistochemistry was performed according to standard protocols (Madhavan *et al*., 2009). Sections were blocked [10% normal goat serum, 0.5% Triton X‐100 in Tris‐buffered saline (TBS)] and incubated in primary antibody overnight at room temperature (RT). Primary antibodies were detected in a 2‐h incubation at RT with secondary antibodies (1:250) coupled to fluorochromes Alexa 488, 594, and 647 (Life Technologies‐Molecular Probes, Grand Island, NY, USA) and counterstained with DAPI. For detecting apoptotic cells, a TUNEL kit (R&D Systems, Minneapolis, MN, USA) was used and manufacturer's instructions followed.

### Statistical analyses

SigmaPlot 11 and GraphPad Prism 6 software were used. For comparing two groups, *t*‐tests were used. For comparisons between three or more groups, analysis of variance (ANOVA) followed by Tukey's or Bonferroni's *post hoc* test for multiple comparisons between treatment groups was conducted. A linear regression analysis as well as a two‐way ANOVA (age factor vs. concentration factor) was used to analyze data from the serial dilution assay. A two‐way repeated‐measures ANOVA was applied to the olfactory behavioral data. Differences were accepted as significant at *P *<* *0.05.

## Author contributions

LM involved in conception and design, collection and assembly of data, and performed data analysis and interpretation, wrote the manuscript, provided financial support, and approved the manuscript; MJC involved in conception and design, collection and assembly of data, and performed data analysis and interpretation, and wrote the manuscript; SR, QWR, BH, and CM involved in collection and assembly of data, and performed data analysis; DDZ provided the study material, involved in experimental design, and wrote the manuscript; and CAB involved in conception and design, provided financial support, and wrote the manuscript.

## Funding

This work was supported by funds from The University of Arizona and an Arizona Biomedical Research Commission (ADHS14‐082982) grant to LM, and the McKnight Brain Research Foundation.

## Conflict of interest

None declared.

## Supporting information


**Fig. S1** NCFC assay, and the characterization of NSPC function and Nrf2 expression under low oxygen conditions.

**Fig. S2** Precise determination of the critical period of decline in NSPC function and Nrf2 expression.

**Data S1** Supplementary methods.
