# Supplementary figures and images for: Reduced Nrf2 expression mediates the decline in neural stem cell function during a critical middle‐age period
Source: Aging Cell. 2016 Apr 20;15(4):725–36. doi: 10.1111/acel.12482 (PMC4933666; doi:10.1111/acel.12482)

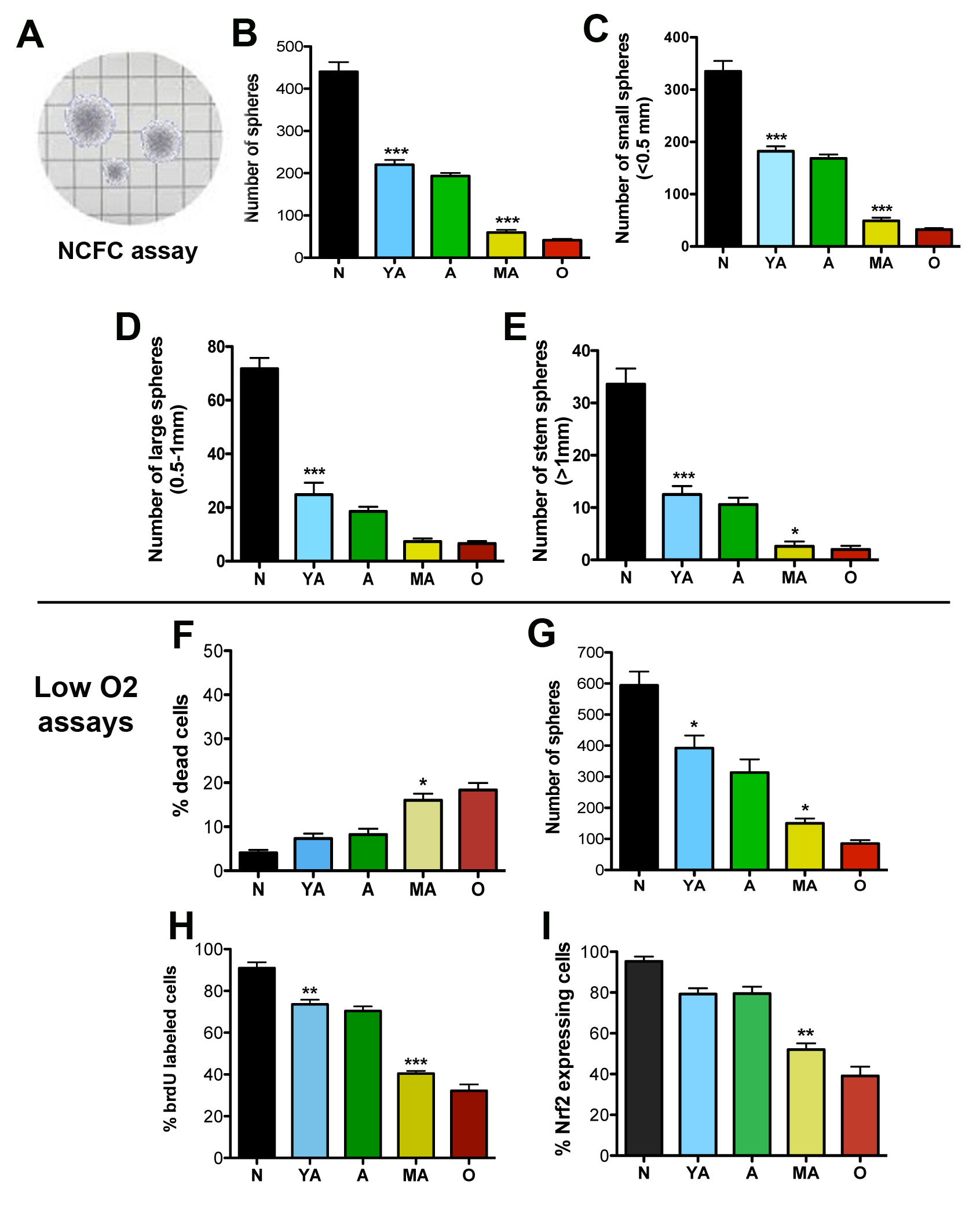

Supplement: Supplementary file 1 — Fig. S1 NCFC assay, and the characterization of NSPC function and Nrf2 expression under low oxygen conditions. [file ACEL-15-725-s001.tif]

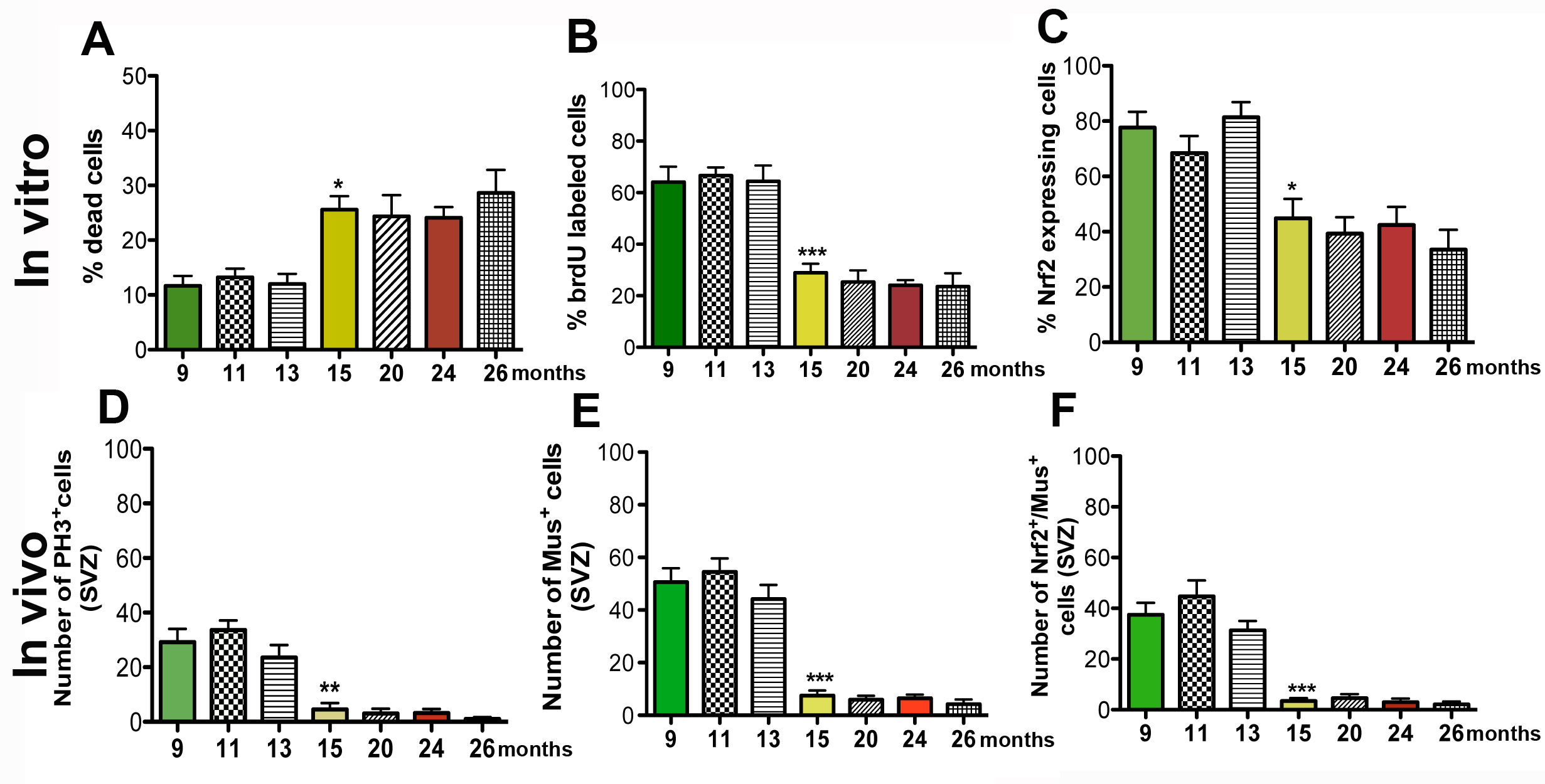

Supplement: Supplementary file 2 — Fig. S2 Precise determination of the critical period of decline in NSPC function and Nrf2 expression. [file ACEL-15-725-s002.tif]
