# Supplementary material for: Reduced Nrf2 expression mediates the decline in neural stem cell function during a critical middle‐age period
Source: Aging Cell. 2016 Apr 20;15(4):725–36. doi: 10.1111/acel.12482 (PMC4933666; doi:10.1111/acel.12482)
Supplement: Supplementary file 3 — Data S1 Supplementary methods. [file ACEL-15-725-s003.docx]

**SUPPLEMENTARY FIGURE LEGENDS**

**Supplementary Figure 1: NCFC assay, and the characterization of NSPC function and Nrf2 expression under low oxygen conditions.** NSPC proliferation was assessed via the NCFC assay (**A**). This analysis showed significant decrements in the total number of neurospheres (**B**), as well as numbers of <0.5mm, 0.5-1mm, and >1.5 mm neurospheres generated in MA and YA cultures, implying that the proliferative function of both stem and progenitor like cells had been negatively impacted at these ages (**C, D, E**). Additionally, NSPCs grown under low oxygen (3% O_2_) were examined via the live-dead (**F**), BrdU (**G**) and neurosphere (**H**) assays. Results indicated a pattern of decline in viability and proliferation comparable to NSPCs grown in ambient laboratory conditions (21% O_2_). Mainly, significant changes were predominantly seen at MA amongst the adult group of cells (at least p<0.05). In addition the percentage of Nrf2 expressing cells also reduced across the age-groups (**I**) and correlated with the data from the viability and proliferation assays. [*p<0.05, **p<0.01, ***p<0.001, one way ANOVAs with post-hoc Tukey’s test].

**Supplementary Figure 2**: **Precise determination of the critical period of decline in NSPC function and Nrf2 expression.** SVZ NSPCs from 11, 13, 20 and 26 mos old rats were also analyzed, in addition to the 5 primary age-groups utilized in the study. These data indicated that *in vitro* the reduction in NSPC survival (**A**, live dead assay, p<0.05 A vs MA) and proliferation (**B**, BrdU assay, p<0.001 A vs MA), and Nrf2 expression (**C**, p<0.05, A vs MA) in fact occurred between 13-15 mos. Viability, proliferative function, and Nrf2 expression of NSPCs from 20 and 26 mos old rats were comparable to MA and O cells. *In vivo*, a similar pattern was observed when a significant reduction in PH3, Mus, Nrf2/Mus double positive cells in the SVZ was noticed at the 15 mos stage (**D-F**, p<0.01, A vs MA). These data support the presence of a critical time window, between 13-15 mos, during which NSPC survival, proliferation, and Nrf2 expression is significantly compromised during aging. [*p<0.05, p<0.001, one way ANOVA with Tukey’s post-hoc test].

**SUPPLEMENTARY METHODS**

**NSPC Isolation and culture:**

*Newborn NSPCs:* Postnatal day 0 rat pups, or Nrf2-/- or WT mice, were anesthetized by subjecting to hypothermia prior to decapitation and brain extraction. SVZs were microdissected and subsequently enzymatically dissociated in 0.05% trypsin-EDTA (*Life Technologies, Grand Island, NY*). Trypsin was subsequently neutralized with Soybean Trypsin Inhibitor (*Sigma-Aldrich, St. Louis, MO*) and cells washed once with Hanks Balanced Salt Solution. Cells were then resuspended in Neurobasal-A Medium containing 1% Glutamax, 2% B-27, 1% Antibiotic-Antimycotic (all from *Life Technologies, Grand Island, NY*), 20 ng/ml EGF, 10 ng/ml bFGF (both from *Cell Sciences, Canton, MA*), and 2 µg/ml of Heparin (*Stemcell Technologies, Vancouver, BC, Canada*). Cells were grown at 37°C under 5% CO_2_ and either 21% O_2_ or low 3% O_2_ for 4-5 days before passaging. 50% of media was replenished every 3 days.

*Adult NSPCs:* Adult rats were subjected to a barbiturate overdose (injected intraperitoneally with 60 mg/kg of Sodium Pentobarbitol (*Sigma-Aldrich, St. Louis, MO*) prior to decapitation and brain extraction. SVZs were microdissected and enzymatically dissociated in a solution of Papain, DNAse, Dispase (PPD: *Worthington Biochemical, Lakewood, NJ; Roche Applied Biosciences, Indianapolis, IN*) for 30 minutes, with trituration. PPD was neutralized with DMEM-F12 (*Life Technologies, Grand Island, NY*) supplemented with 10% Fetal Bovine Serum (FBS, *Atlanta Biologicals, Norcross, GA*), and the cell pellet was washed twice, first with DMEM-F12/FBS and then DMEM-F12. After final centrifugation, adult NSPCs were resuspended in Neurobasal-A Medium containing 1% Glutamax, 2% B-27, 1% Antibiotic-Antimycotic, 20 ng/ml EGF, 20 ng/ml bFGF, and 2 μg/ml of Heparin. Cells were maintained at 37°C under 5% CO_2_ and either 21% O_2_ or low 3% O_2_ for 7-10 days before passaging. 50% of media was replenished every 3 days.

**NSPC Differentiation:** NSPCs from each age group of rats, or WT or Nrf2-/- mice, were enzymatically dissociated and plated onto Poly-D-Lysine (0.1 mg/ml, *Sigma-Aldrich, St. Louis, MO*) and Laminin (10 μg/ml, *Sigma-Aldrich, St. Louis, MO*) coated glass coverslips placed in 24 well plates at a density of 30,000 cells/well. To induce differentiation, growth factors were retrieved and cells were maintained in medium consisting of Neurobasal-A with 1% Glutamax, 2% B-27, 1% Antibiotic-Antimycotic, and 2% Fetal Bovine Serum (FBS, *Atlanta Biologicals, Norcross, GA*). Differentiation into neuronal and glial cell types was assessed via immunocytochemistry after 10 days in culture. 5 fields per coverslip were assessed and the percentage of Dapi stained cells expressing Tuj1 (neurons), S100β (astrocytes) or RIP (oligodendrocytes) were counted under a 20X lens.

The Neural Colony Forming Cell (NCFC) Assay was carried out using the NeuroCult Rat NCFC Assay Kit (*Stemcell Technologies, Vancouver, BC*) according to manufacturer instructions. In this assay, NSPCs were grown in a semi-solid medium to support their growth as independent clonal populations (colonies). Larger colonies are presumably derived from stem cells, which have a greater potential to divide and generate daughter cells, wherein smaller colonies are likely produced by neural progenitor cells. Briefly, NSPCs were plated at a density of 5 x10^3^ cells per 35mm dish in a collagen matrix using a proliferation medium consisting of NeuroCult basal medium, with supplements (EGF, bFGF, and 0.2% Heparin). Each dish was supplemented twice a week with the proliferation medium, and maintained for 3 weeks. Subsequently, using a scoring dish with a 2 x 2 mm grid, the number of neurospheres of varying sizes (<0.5 mm, 0.5-1 mm, and >1 mm) were enumerated in every culture dish under a 2.5X objective.

**NSPC survival (Live-Dead Assay):** NSPC viability was assessed using a Live-Dead cell assay kit (*Life Technologies, Grand Island, NY*) according to the manufacturer instructions. Briefly, 30,000 cells were plated onto glass coverslips coated with Poly-D-Lysine/Laminin in 24 well plates using proliferation medium. Media was subsequently removed and the cells exposed to 4 μM Ethidium Homodimer 1 and 2 μM Calcein AM dye in 1X D-PBS (*Life Technologies, Grand Island, NY*). After 45 minutes, the number of green cells (live, labeled with Calcein AM dye) and red cells (dead, labeled with Ethidium Homodimer 1) were counted in 5 random fields per coverslip under a 20X lens.

**siRNA and Transfection assays:** NSPCs from newborn (P0) or middle-aged (15 mos) rats were plated at a density of 25,000 cells/well in 24 well plates. For the knock-down studies, the cells were treated with siRNAs (*Santa Cruz Biotechnology, Dallas, TX*) targeting Nrf2, control siRNAs, or PBS. The transfections occurred when the cells were 70-80% confluent using Lipofectamine RNAiMAX Transfection reagent (*Life Technologies, Grand Island, NY*) by following the manufacturers instructions. After 48 hours, the medium was restored to basic NSPC proliferation medium, and cells assessed via immunohistochemistry, live-dead assay, or BrdU labeling. For the overexpression studies, a rat Nrf2 expression plasmid (CMV promoter, *Creative Biogene Technology, Shirley, NY*) was used and transfected into NSPCs using Lipofectamine LTX reagent (*Life Technologies, Grand Island, NY*) following protocols from the manufacturer. NSPCs were initially plated at 25,000 cells/well in 24 well plates, and the transfections started when the cultures were 70-80% confluent. Parallel NSPC cultures treated with only Lipofectamine LTX reagent served as controls. After 72 hours, transfection medium was removed and proliferation medium added. The transfected and control NSPCs were then analyzed via immunohistochemistry, live-dead and BrdU assays.

**Fine Olfactory Discrimination:** The task included an initial training and subsequent testing stages for Discrete and Fine Olfactory Discrimination abilities after water restriction for 48 hours (a 1 hour period of access to water/day was allowed).

*Training:* 12 uL of double distilled water with 1 uL of coconut extract (COC) was placed in a sterile dish – a combination which served as a reward designated [+]. The dish was placed at one end of the cage, and the animal was given 2 min to find and drink the [+]. The dish was removed once the animal had finished drinking, and after a 30 sec interval, the animal was again presented with the dish containing fresh [+] solution. Each trial involved an increase in the amount of COC until it reached 8.5 uL per dish per trial. From here, five additional trials were conducted using [+]. For the sixth trial, animals received culture dishes containing 8.5 uL of almond extract (ALM) applied mixed with 12 uLs of 1% denatonium benzoate (DB) (Sigma-Aldrich, St. Louis, MO). This combination of ALM and DB is extremely bitter and was designated [-]. The animals naturally found the [-] aversive, learned to associate the bitter taste with the smell of ALM, and subsequently avoided drinking the [-]. Four additional trials were conducted with [-] to ensure that the animals had learned to avoid the bitter liquid.

*Discrete discrimination testing:* In the testing stage, animals were presented with two dishes, one containing [+], and the other containing [-]. In this situation, the animals were given 2 mins to select a dish to drink from. Similar to the training period, after a 30 sec inter-trial interval, the animals were presented with the dishes containing [+] and [-] solutions again. A successful discrimination constituted a animal drinking the [+]. The criteria for failure was the following: (1) the animal chose the [-] rather than the [+]; (2) the animal chose the [+] but within 30 sec of that selection went on to drink the [-], indicating that they could not effectively discriminate between the two; (3) the animal made no choice in the allocated 2 mins. In case of a failure, both dishes were immediately removed from the cage, concluding the trial. This occurred for a total of 10 trials. The positions of [+] and [-] dishes were randomized from trial to trial to prevent the animals from making selection decisions based on location. In addition, two extra error-checking (unrecorded) trials in which both dishes contained only [+] were also conducted during the testing, so as to prevent a high-performing animal from ignoring the second dish after successfully drinking from the first. Hence the animals were trained to believe that there were always two possible solutions available in any trial ensuring that they would sniff at the [-] even after correctly tasting the [+] (basically a variable reinforcement schedule).

*Fine discrimination testing:* In this testing scenario, the COC [+] and ALM [-] dishes were replaced with dishes that contained various ratios of COC and ALM. In this situation, the ability of the experimental animals to distinguish between solutions containing [+] ratios of COC:ALM of 100:0 (basic discrete discrimination), 60:40, 58:42, and 56:44 as well as the corresponding [-] ratios of COC:ALM of 0:100, 40:60, 42:58 and 44:56 was tested. Every trial utilizing a certain mixture of [+] and [-] mixture was conducted with the same 2 min time limit and 30 sec inter-trial interval. This was repeated until a total of 5 trials were conducted for each concentration tested.

**Primary antibody details:**

| **Antibody** | **Vendor** | **Catalog #** | **Host/Type** |
| --- | --- | --- | --- |
| anti-Bromodeoxyuridine, IgG2a BU1/75 (ICR1) | Abcam | ab6326 | Rat/Monoclonal |
| anti-Nestin, Clone: rat-401 | Millipore | MAB353 | Mouse/Monoclonal |
| anti-Neuronal Class III Beta-Tubulin (TUJ1) | Covance | MRB-435P | Rabbit/Monoclonal |
| anti-S100 beta-subunit, Clone: SH-B1 | Sigma-Aldrich | S2532 | Mouse/Monoclonal |
| anti-Oligodendrocytes (RIP) , Clone: NS-1 | Millipore | MAB1580 | Mouse/Monoclonal |
| anti-Nrf2, C-20 | Santa Cruz | SC-722 | Rabbit/Polyclonal |
| anti-Nrf2, H-300 | Santa Cruz | SC-13032 | Rabbit/Polyclonal |
| anti-Phospho-Histone H3 (Ser10) | Cell Signaling | 9701 | Rabbit/Polyclonal |
| anti-Glial Fibrillary Acidic Protein (GFAP), Clone: GA5 | Millipore | MAB360 | Mouse/Monoclonal |
| anti-Musashi-1 | Millipore | AB5977 | Rabbit/Polyclonal |
| anti-Ki67 [SP6] | Abcam | ab16667 | Rabbit/Monoclonal |
| anti-SOX2 | Abcam | ab97959 | Rabbit/Polyclonal |
| Anti-χGCSm (E-4) | Santa Cruz | sc-55586 | Mouse/Monoclonal |
| Anti-NQO1 (A180) | Santa Cruz | sc-32793 | Mouse/Monoclonal |
| Anti-HO1 (A-3) | Santa Cruz | sc-136960 | Mouse/Monoclonal |

*Immunocytochemistry* - The primary antibodies concentrations used were as follows: Nestin (1:300) and Oligodendrocytes RIP (1:500) from EMD Millipore, Billerica MA; Neuronal Class III Beta-Tubulin (TUJ1, 1:300: Covance, Princeton, NJ); S-100β-Subunit 1 (1:1000, Sigma-Aldrich, St. Louis, MO); Nrf2-C20 (1:200), Nrf2-H300 (1:200), GCLM (1:200), NQO1 (1:200), and HO1 (1:200) from Santa Cruz Biotechnology, Dallas, TX.

*Immunohistochemistry* – The primary antibody concentrations applied were as follows: Nrf2-C20 (1:100), Nrf2-H300 (1:100) GCLM (1:200), NQO1 (1:200), and HO1 (1:200) from Santa Cruz Biotechnology, Dallas, TX; Phospho-Histone H3 (Ser10) (PH3: 1:100, Cell Signaling Technology, Danvers, MA); Musashi-1 (1:300) and Glial Fibrillary Acidic Protein (GFAP Clone-GA5: 1:500) and Dcx (1:500) from EMD Millipore, Billerica MA; Ki67 (1:500) and Sox2 (1:500) from Abcam, Cambridge, MA.

Control conditions constituted the deletion of the primary antibody or secondary antibody and the inclusion of relevant isotype specific antibodies and sera instead of the omitted antibodies.
